# Prognostic Performance of the Korean Triage and Acuity Scale Combined with the National Early Warning Score for Predicting Mortality and ICU Admission at Emergency Department Triage: A Retrospective Observational Study

**DOI:** 10.3390/diagnostics16020345

**Published:** 2026-01-21

**Authors:** Jungtaek Park, Sang Hoon Oh, Ae Kyung Gong, Jee Yong Lim, Sun Hee Woo, Won Jung Jeong, Ji Hoon Kim, In Soo Kim, Soo Hyun Kim

**Affiliations:** 1 Department of Emergency Medicine, Uijeongbu St. Mary’s Hospital, College of Medicine, The Catholic University of Korea, Seoul 06591, Republic of Korea; trainfunnel@gmail.com; 2 Department of Emergency Medicine, Seoul St. Mary’s Hospital, College of Medicine, The Catholic University of Korea, Seoul 06591, Republic of Korea; kyung6069@naver.com (A.K.G.); ny1117@gmail.com (J.Y.L.); 3 Department of Emergency Medicine, Incheon St. Mary’s Hospital, College of Medicine, The Catholic University of Korea, Seoul 06591, Republic of Korea; drme@catholic.ac.kr; 4 Department of Emergency Medicine, Suwon St. Vincent Hospital, College of Medicine, The Catholic University of Korea, Seoul 06591, Republic of Korea; medpooh@catholic.ac.kr; 5 Department of Emergency Medicine, Bucheon St. Mary’s Hospital, College of Medicine, The Catholic University of Korea, Seoul 06591, Republic of Korea; intimator@naver.com; 6 Department of Emergency Medicine, Daejeon St. Mary’s Hospital, College of Medicine, The Catholic University of Korea, Seoul 06591, Republic of Korea; kkis329@naver.com; 7 Department of Emergency Medicine, Eunpyeong St. Mary’s Hospital, College of Medicine, The Catholic University of Korea, Seoul 06591, Republic of Korea; unidgirl@catholic.ac.kr

**Keywords:** emergency department, triage, early warning score, ICU admission, mortality

## Abstract

**Objectives**: This study aimed to compare the predictive performance of the Korean Triage and Acuity Scale (KTAS) and the National Early Warning Score (NEWS) for serious adverse events (SAEs), including mortality and intensive care unit (ICU) admission, during emergency department (ED) stay. We also evaluated whether combining the two systems improves prediction accuracy. **Methods:** This retrospective study included adult patients (≥19 years) who presented to a university-affiliated ED between October and December 2024. KTAS and NEWS were assessed simultaneously at triage. NEWS2 was calculated retrospectively based on routinely documented vital signs and medical history without performing routine arterial blood gas analysis. The primary outcome was the occurrence of SAE during the ED stay. Predictive performance was evaluated using receiver operating characteristic (ROC) curves and the area under the curve (AUC), and logistic regression models were used to identify independent associations. **Results:** A total of 4216 patients were analyzed, of whom 255 (6.0%) experienced SAEs. All three scores—KTAS, NEWS and NEWS2—were independently associated with the occurrence of SAEs. The AUCs for KTAS, NEWS and NEWS2 were 0.75 (95% CI, 0.74–0.76), 0.73 (95% CI, 0.71–0.74) and 0.73 (95% CI, 0.71–0.74), respectively. Combining KTAS with NEWS or NEWS2 significantly improved predictive accuracy (AUC 0.81, 95% CI 0.79–0.82; *p* < 0.001). **Conclusions:** Both KTAS and NEWS/NEWS2 reliably predicted in-ED adverse outcomes, and their combination further enhanced prognostic performance. Integrating physiology-based early warning scores with structured triage systems may help identify high-risk ED patients earlier and optimize resource allocation.

## 1. Introduction

Over the past decades, the global increase in emergency department (ED) visits has contributed to overcrowding and resource constraints, ultimately delaying timely and appropriate emergency care [1]. In addition, population aging and the rising prevalence of chronic diseases have led to an increasing proportion of ED patients presenting with multimorbidity and atypical clinical features. These trends complicate the identification of high-risk patients during initial triage, particularly those prone to serious adverse events (SAEs) such as mortality or intensive care unit (ICU) admission, which reflect clinically meaningful deterioration during their ED stay.

In South Korea, ED crowding has been recognized as a major healthcare challenge, with nationwide analyses demonstrating that delays in ED care are associated with increased patient deterioration and adverse outcomes [2,3]. In response to the need for appropriate and timely risk stratification of emergency patients within the local healthcare context, the Canadian Triage and Acuity Scale (CTAS) was adapted to develop the Korean Triage and Acuity Scale (KTAS), which has since been implemented nationwide [4,5]. To prioritize treatment, the KTAS categorizes patients into five levels of acuity based on presenting complaints and vital signs, reflecting the urgency and severity of their condition [4,5]. However, like other complaint-based triage systems, KTAS prioritizes sensitivity to avoid under-triage, which may result in substantial over-triage and limited specificity. This characteristic, while appropriate for patient safety, can complicate real-time identification of patients with subtle or evolving physiological deterioration, particularly in crowded ED settings.

Early warning scores (EWS), such as the National Early Warning Score (NEWS) and its updated version NEWS2, were developed to detect acute physiological deterioration [6,7]. The NEWS incorporates six routinely measured physiological parameters—systolic blood pressure, heart rate, respiratory rate, body temperature, peripheral oxygen saturation (SpO_2_), and level of consciousness—allowing for simple application with minimal training requirements [8]. Previous studies have evaluated the clinical utility of NEWS across various clinical settings [9,10,11,12,13,14] and demonstrated that NEWS provides an objective, physiology-based assessment of risk for cardiac arrest and unanticipated ICU admission [15,16]. Importantly, NEWS can be calculated rapidly using data already obtained during routine triage, without requiring additional tests or delaying clinical decision-making. Nevertheless, when applied alone at a single time point in the ED, NEWS may insufficiently capture contextual factors related to presenting complaints, anticipated resource needs, or triage urgency.

Because triage tools and EWSs are designed to assess patient risk from fundamentally different perspectives—clinical urgency versus physiological instability—they may provide complementary rather than redundant information. Despite this theoretical complementarity, limited data exist evaluating whether integrating KTAS with NEWS or NEWS2 at the time of ED triage provides incremental and clinically meaningful prognostic value beyond either tool alone. Therefore, the present study aimed to evaluate the predictive performance of KTAS and NEWS for SAEs, such as mortality and ICU admission during the index ED visit and to determine whether combining KTAS with NEWS-based scores at triage improves risk stratification.

## 2. Materials and Methods

### 2.1. Study Design and Setting

We conducted a retrospective observational study at a university-affiliated ED between October and December 2024. All adult patients (≥19 years) presenting during the study period were eligible for inclusion; therefore, no formal sample size calculation was performed. Patients were excluded if they presented with cardiac arrest at the scene or on arrival, visited for non-medical reasons, left against medical advice, or had missing data. The requirement for written patient consent was waived by the Institutional Review Board of the Catholic University of Korea, Seoul St. Mary’s Hospital (KC25RISI0806), in accordance with local legal regulations, because this retrospective study analyzed anonymized medical records. The study was conducted and reported in accordance with the Strengthening the Reporting of Observational Studies in Epidemiology guidelines (Table S1) [17].

### 2.2. KTAS and NEWS Assessment

In South Korea, ED patient information—including demographics, arrival mode, vital signs, KTAS level, diagnoses, and dispositions—is uniformly managed through the nationwide National Emergency Department Information System (NEDIS) [18]. All triage nurses are trained and certified in KTAS assessment. KTAS levels are determined based on presenting complaints, vital signs, and first/second modifiers such as pain score, hemorrhagic conditions, mechanism of injury, blood glucose level, or dehydration [4,5]. The KTAS assigns five triage levels that determine the maximum recommended waiting time until physician assessment: level 1 (resuscitation), immediately; level 2 (emergent), within 30 min; level 3 (urgent), within 60 min; level 4 (less urgent), within 90 min; and level 5 (non-urgent), within 120 min.

Since November 2021, NEWS has been automatically calculated within our electronic medical record system using vital signs that are routinely measured and entered during ED triage. In contrast, NEWS2 requires arterial blood gas analysis (ABGA) to determine the appropriate SpO_2_ scale, which is not routinely available at the time of triage. Accordingly, NEWS2 scores were not calculated prospectively at triage but were retrospectively derived through medical chart review, which may have introduced some degree of misclassification. For the adjustment of hypercapnic respiratory failure required for NEWS2 SpO_2_ Scale 2, documented medical history—including chronic obstructive pulmonary disease (COPD), obesity hypoventilation syndrome, neuromuscular disorders, or severe thoracic deformities—was used as surrogate criteria [19]. Both NEWS and NEWS2 assigns 0–3 points to six parameters, with an additional point for supplemental oxygen. The total score stratifies patients into low (0–4), medium (5–6), or high risk (≥7) [20]. Patients with NEWS ≥ 7 were immediately assessed by emergency physicians, regardless of KTAS level.

### 2.3. Variables and Data Collection

Baseline characteristics, visit time, disease type, arrival mode, referral routes, vital signs, KTAS levels, and outcomes were extracted from NEDIS. Visit time was categorized as daytime or nighttime/weekend. Disease type was classified as medical or trauma. Arrival mode was categorized as ambulance or walk-in. Referral routes were classified as direct visit, outpatient referral, or transfer from another facility.

### 2.4. Outcomes

The primary outcome was SAE during the ED stay, defined as in-ED mortality or ICU admission. Patients who underwent surgery and were admitted to the ICU, as well as those transferred to ICUs in other facilities due to ICU unavailability, were included in the SAE group.

### 2.5. Statistical Analysis

Categorical variables were presented as counts and percentages, continuous variables as mean ± standard deviation or medians with interquartile ranges (IQR) as appropriate. Group differences were assessed using the chi-squared test for categorical variables and Student’s t-test or the Mann–Whitney U test for continuous variables. Receiver operating characteristic (ROC) curves were generated, and predictive performance was evaluated using the area under the ROC curve (AUC) with 95% confidence intervals (CI). We further examined the predictive performance of widely used categorical thresholds for both KTAS (e.g., ≤3 and ≤2) and NEWS/NEWS2 (medium- and high-risk categories) by calculating corresponding sensitivities and specificities. Pearson correlation analysis was used to examine the association between KTAS and NEWS or NEWS2.

Additionally, logistic regression analyses were performed to examine associations between clinical scales and outcomes. KTAS level and NEWS/NEWS2 were entered into the models as independent continuous variables without interaction terms, and models were adjusted for potential confounders. To evaluate the predictive performance of each model (KTAS and NEWS or KTAS and NEWS2), predicted probabilities derived from the logistic regression models were used to generate ROC curves and to calculate the AUC values. Pairwise AUC comparisons were conducted using the DeLong method [21]. Analyses were performed using SPSS version 24 (IBM, Armonk, NY, USA) and MedCalc version 12 (MedCalc Software, Mariakerke, Belgium). A *p*-value < 0.05 was considered significant.

## 3. Results

### 3.1. Baseline Characteristics of Participants

**Figure 1:**
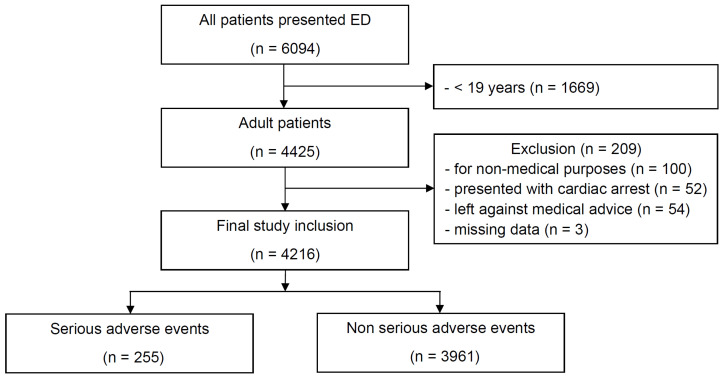
Flowchart of patient inclusion and exclusion in the study. ED, emergency department.

A total of 6094 adult patients presented to our ED during the study period. Of these, 1878 patients were excluded for the following reasons: 1669 patients were younger than 19 years, 100 visited for non-medical purposes, 52 were confirmed to have cardiac arrest at the scene or upon ED arrival, 54 left the ED against medical advice, and 3 had missing data. Finally, 4216 patients were included in the analysis (Figure 1), of whom 2119 (50.3%) were male and 2097 (49.7%) were female. The mean age of participants was 60.8 ± 18.1 years. The median KTAS and NEWS were 3 (IQR, 3–3) and 1 (IQR, 0–3), respectively (Table 1). The SpO_2_ scale 2 of the NEWS2 was applied in 79 patients based solely on a documented previous history, and the median NEWS2 was 1 (IQR, 0–3).

SAEs occurred in 255 patients (mortality, 13; ICU admission, 242). Baseline characteristics of the study population, stratified by outcome, are presented in Table 1. Patients in the SAE group were significantly older and more frequently male compared to those without SAE (65.2 ± 15.5 vs. 60.5 ± 18.2, *p* < 0.001; 151 (59.2) vs. 1968 (49.7), *p* = 0.003). KTAS and NEWS/NEWS2 scores also differed significantly between the SAE and non-SAE groups (KTAS: 2 [IQR, 2–3] vs. 3 [IQR, 3–3], *p* < 0.001; NEWS: 3 [IQR, 1–6] vs. 1 [IQR, 0–2], *p* < 0.001; NEWS2: 3 [IQR, 1–6] vs. 1 [IQR, 0–2], *p* < 0.001). The SAE group had a higher proportion of patients transferred from other facilities and those who arrived by ambulance. No significant differences were observed in disease type or time of visit between the groups. Pearson correlation analysis revealed a moderate negative correlation between KTAS and NEWS (or NEWS2) (r = −0.413, *p* < 0.001; r = −0.427, *p* < 0.001), whereas NEWS and NEWS2 values were near-identical (r = 0.983, *p* < 0.001).

### 3.2. KTAS and NEWS/NEWS2 for Predicting SAE

**Figure 2:**
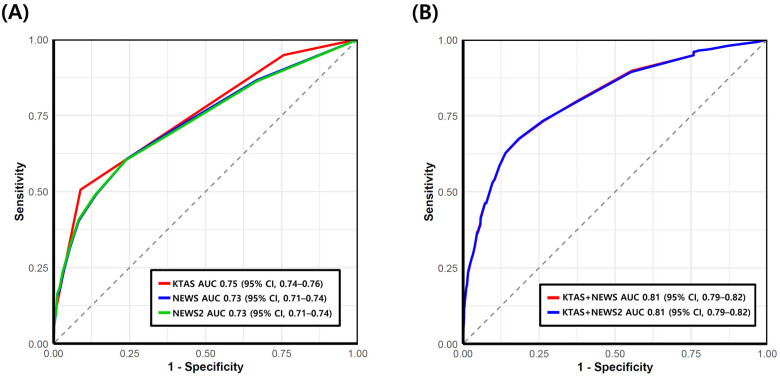
Receiver operating characteristic (ROC) curves of the Korean Triage and Acuity Scale (KTAS), National Early Warning Score (NEWS) and NEWS2 for predicting serious adverse events (**A**), and combined models of KTAS with NEWS or NEWS2 (**B**).

The predictive value of each scale was assessed using ROC analysis (Figure 2A). KTAS demonstrated an AUC of 0.75 (95% CI, 0.74–0.76). Using a cut-off of level ≤ 3, sensitivity and specificity for SAE were 94.9% (95% CI, 91.4–97.3) and 24.3% (95% CI, 23.0–25.7), respectively. Using a cut-off of ≤2, sensitivity was 50.6% (95% CI, 44.3–56.9) and specificity was 91.1% (95% CI, 90.2–92.0) (Table 2).

NEWS and NEWS2 showed comparable prognostic performance, with an AUC of 0.73 (95% CI, 0.71–0.74). A NEWS value of ≥5 (medium risk) predicted SAE with 40.4% (95% CI, 34.3–46.7) sensitivity and 91.7% (95% CI, 90.8–92.5) specificity, whereas a cut-off of ≥7 (high risk) yielded 23.5% sensitivity (95% CI, 18.5–29.2) and 97.0% specificity (95% CI, 96.4–97.5). Given the near-identical values of NEWS and NEWS2 in this cohort, their sensitivities and specificities were essentially the same (Table 2).

Univariate analysis demonstrated that lower KTAS levels (odds ratio [OR], 0.17 [95% CI, 0.14–0.21]) and higher NEWS or NEWS2 scores (OR, 1.39 [95% CI, 1.33–1.45] for both) were associated with SAE (Table 3). Additionally, male gender, older age, ambulance use, and referral route were significantly associated with SAE. In multivariate analyses, adjusting for potential confounders, lower KTAS and higher NEWS or NEWS2 remained independent predictors of SAE (adjusted OR [AOR], 0.33 [95% CI, 0.26–0.42]; AOR, 1.18 [95% CI, 1.12–1.24] for both NEWS and NEWS2).

### 3.3. Prognostic Performance of KTAS Combined with NEWS or NEWS2

When KTAS was combined with NEWS or NEWS2 in logistic regression models, the predictive accuracy for SAE significantly improved (Figure 2B). The AUC increased to 0.81 (95% CI, 0.79–0.82) for both combinations. Compared with KTAS alone, these differences were statistically significant (all *p* < 0.05). Given the close similarity of NEWS and NEWS2 in this population, either score could be used interchangeably for combination models without meaningful loss of accuracy.

To further illustrate the clinical implications of combining KTAS with NEWS, we analyzed the distribution of SAE according to NEWS categories (NEWS ≥ 5 vs. <4) within patients triaged as KTAS level 3 (Table 4). Among these patients, a NEWS ≥ 5 was associated with a significantly higher incidence of SAEs compared with lower NEWS categories (*p* < 0.001).

## 4. Discussion

In this study, we evaluated the predictive performance of KTAS and NEWS/NEWS2 for the occurrence of SAEs during the ED stay. Our results demonstrated that KTAS and NEWS/NEWS2 had comparable prognostic ability for predicting SAEs. However, models combining KTAS with NEWS or NEWS2 at triage showed superior performance compared with either KTAS or NEWS/NEWS2 alone.

Our finding that KTAS level predicts mortality or ICU admission in ED patients aligns with previous studies [22,23,24]. KTAS has been validated in multiple clinical investigations and has been reported to perform well in the early identification of ED patients at increased risk of mortality, consistent with studies using CTAS [25]. Lim et al. reported that lower KTAS levels were associated with higher 30-day mortality in severe trauma, and its predictive ability for medical conditions has also been demonstrated [22,23]. These findings support the reliability of KTAS as a triage tool for risk stratification in heterogeneous ED populations [24]. Notably, more than half of our patients (*n* = 1789, 51.3%) were classified as KTAS level 3, and using this cut-off as the urgent category demonstrated high sensitivity but low specificity for SAE occurrence, consistent with prior reports suggesting that KTAS may overestimate patient severity [26].

In the present study, implementation of NEWS at triage was feasible, as it incorporates routinely measured vital signs [6], and it provided additional value in identifying high-risk patients without increasing workload. NEWS was originally designed to objectively monitor patient status over time, indicating improvement or deterioration. Its prognostic utility has been well established in ward patients and in patients transferred with clinical deterioration, particularly for predicting ICU admission [15,27,28]. However, in the ED setting, prior studies have often been limited to small or pre-selected cohorts [14,29,30,31]. Although NEWS has been increasingly evaluated in all ED populations [16,32,33,34], the value of a single initial measurement at triage has not been firmly established. Unlike ward patients, ED patients may already be in a deteriorated state or receive immediate interventions that alter their clinical course. Moreover, outcomes of interest to emergency physicians, such as ED discharge status, have not been thoroughly studied in heterogeneous populations with proper adjustment for confounders. Our results indicate that measuring NEWS at triage is useful for identifying patients at risk of SAEs during ED stay, with particularly good specificity.

NEWS2 was developed to improve specificity in patients with hypercapnic respiratory failure [7,35,36]. However, its potential incremental value in ED triage remains of clinical interest. Accordingly, we evaluated parallel models incorporating either NEWS or NEWS2 to assess whether this theoretical advantage translated into improved prognostic performance in a real-world, unselected ED population. In our study, however, NEWS2 did not significantly outperform NEWS, and replacing NEWS with NEWS2 did not yield clinically meaningful improvement. This finding is consistent with prior reports indicating that NEWS2 does not consistently provide additional prognostic value compared with NEWS in general ED populations, except in selected subgroups such as patients with chronic hypoxemia or COPD [16,37]. Potential explanations include subgroup effects, variability in outcome definitions and observation windows, and differences in oxygen supplementation practices. Furthermore, using NEWS2 at a single time point rather than assessing its trajectory may limit its predictive value [38,39]. In our cohort, ABGA was not routinely obtained at triage; therefore, NEWS2 SpO_2_ Scale 2 was assigned solely on the basis of documented prior medical history. This approach may have resulted in misclassification of some patients, which could partly explain the minimal performance difference between NEWS and NEWS2. However, from a practical perspective, these results support the use of NEWS as a simpler and sufficiently robust tool for integration with KTAS at triage, without requiring additional assumptions related to hypercapnic respiratory failure.

Previous reports have compared triage tools with EWSs such as NEWS, NEWS2, and the modified early warning score in ED populations [33,40]. EWSs generally assess similar aspects of physiological deterioration, whereas triage tools evaluate urgency and resource requirements, offering complementary rather than competing information. Combining a complaint-based triage scale with a physiology-based EWS may help preserve the high sensitivity of triage systems while improving specificity and identification of patients with masked or evolving deterioration. Our findings demonstrate that integrating NEWS or NEWS2 into KTAS improved the prediction of SAEs. Although KTAS ≤ 3 demonstrated high sensitivity for SAE, its low specificity resulted in a substantial number of false-positive triage alerts. When NEWS was applied specifically within the KTAS level 3 (urgent) group, higher NEWS values (≥5, medium to high risk) were associated with a markedly increased risk of SAE, suggesting that NEWS may serve as an effective physiological filter to improve specificity without compromising the safety provided by KTAS. Given that NEWS parameters are routinely obtained as part of triage, combining KTAS with NEWS is both practical and potentially beneficial across a variety of clinical settings.

This study has several limitations. First, although NEWS was prospectively measured at triage alongside KTAS, the study design was retrospective. NEWS results were not blinded to physicians and may have partially influenced the timing of physician encounters and decisions regarding ICU admission or mortality. Second, NEWS2 was retrospectively calculated, and hypercapnic respiratory failure was assumed based solely on past medical history without arterial blood gas data. These factors may have introduced misclassification bias. Acute confusion, an element of NEWS2 that could affect performance, was also not evaluated. These limitations should be considered when interpreting the comparable performance observed between NEWS and NEWS2 in our cohort. Third, although the overall sample size was substantial, the number of SAEs was relatively low, reflecting the real-world incidence of mortality and ICU admission in an unselected ED population. Consequently, the ratio of events to predictors may raise concerns regarding model robustness and potential overfitting. Furthermore, our prediction models were constructed without internal or external validation. Fourth, the study was conducted in South Korea, where KTAS is standard; thus, generalizability to healthcare systems using other triage scales (e.g., CTAS, Japan Triage Acuity Scale) may be limited. Therefore, future studies should validate these findings in independent, multicenter cohorts and explore prospective implementation to assess reproducibility and clinical impact in diverse settings. Nonetheless, our findings highlight that combining NEWS with KTAS can substantially improve prediction of mortality and ICU admission without increasing workload.

## 5. Conclusions

In this retrospective single-center study, both KTAS and NEWS independently predicted mortality and ICU admission among ED patients. Their combination improved risk stratification at triage, potentially facilitating earlier detection of masked clinical deterioration and more efficient ICU resource utilization. Given that NEWS is automatically derived from routinely collected vital signs and NEWS2 offered no meaningful incremental benefit, integrating KTAS with physiology-based NEWS represents a practical and scalable approach for electronic health record-based triage systems.

## Figures and Tables

**Table 1:** Baseline characteristics of study cohort according to the occurrence of serious adverse events.

	Total Patients (*n* = 4216)	SAE Group (*n* = 255)	Non-SAE Group (*n* = 3961)	*p*-Value
Male	2119 (50.3)	151 (59.2)	1968 (49.7)	0.003
Age, years, mean ± SD	60.8 ± 18.1	65.2 ± 15.5	60.5 ± 18.2	<0.001
Disease type				
Medical	3879 (92.0)	240 (94.1)	3639 (91.9)	0.200
Trauma	337 (8.0)	15 (5.9)	322 (8.1)	
Referral route				<0.001
Outpatient department	345 (8.2)	11 (4.3)	334 (8.4)	
Direct visit	3170 (75.2)	162 (63.5)	3008 (75.9)	
Transfer from another hospital	701 (16.6)	82 (32.2)	619 (15.6)	
Mode of visit				
Walk-in	3010 (71.4)	82 (32.2)	2928 (73.9)	<0.001
Ambulance	1206 (28.6)	173 (67.8)	1033 (26.1)	
Visit time				
Daytime	1814 (43.0)	108 (42.4)	1706 (43.1)	0.823
Night/weekend	2402 (57.0)	147 (57.6)	2255 (56.9)	
KTAS, median (IQR)	3 (3 –3)	2 (2–3)	3 (3–3)	<0.001
NEWS, median (IQR)	1 (0–3)	3 (1–6)	1 (0–2)	<0.001
NEWS2, median (IQR)	1 (0–3)	3 (1–6)	1 (0–2)	<0.001
Hypercapnic respiratory failure	78 (1.9)	7 (2.7)	71 (1.8)	0.274
Mean blood pressure, mmHg, mean ± SD	95.7 ± 18.4	93.6 ± 25.6	95.8 ± 17.8	0.169
Heart rate, per min, mean ± SD	88.4 ± 20.0	89.8 ± 25.0	88.3 ± 19.6	0.348
Respiratory rate, per min, mean ± SD	19.3 ± 2.4	20.0 ± 3.2	19.3 ± 2.3	<0.001
Body temperature, mean ± SD	36.8 ± 0.8	36.6 ± 0.9	36.8 ± 0.8	0.001
O2 supplement	269 (6.4)	78 (30.6)	191 (4.8)	<0.001
Saturation, mean ± SD	96.9 ± 3.5	95.5 ± 5.5	97.0 ± 3.2	<0.001
Mentality				<0.001
Alert	4021 (95.4)	189 (74.1)	3832 (96.7)	
Verbal	76 (1.8)	30 (11.8)	46 (1.2)	
Unresponsive	38 (0.9)	22 (8.6)	16 (0.4)	
Pain	81 (1.9)	14 (5.5)	67 (1.7)	
ED LOS, h, median (IQR)	3.8 (2.2–8.1)	3.5 (1.9–6.6)	3.8 (2.2–8.2)	0.040

Data are presented as *n* (%) for the categorical variables unless otherwise indicated. ED LOS was the time from ED arrival to hospitalization or discharge. SAE, serious adverse event; SD, standard deviation; IQR, interquartile range; KTAS, Korean Triage and Acuity Scale; NEWS, National Early Warning Score; ED; emergency department; LOS, length of emergency department stay.

**Table 2:** Prognostic performance of KTAS, NEWS, and NEWS2 for predicting mortality and intensive care unit admission during the index emergency department visit.

	AUC (95% CI)	Cut-Off Value	Sensitivity (95% CI)	Specificity (95% CI)	*p*-Value
KTAS	0.75 (0.74–0.76)	≤3	94.9 (91.4–97.3)	24.3 (23.0–25.7)	<0.001
		≤2	50.6 (44.3–56.9)	91.1 (90.2–92.0)	
NEWS	0.73 (0.71–0.74)	≥3	60.8 (54.5–66.8)	75.8 (74.4–77.1)	<0.001
		≥4	49.0 (42.7–55.3)	86.0 (84.9–87.1)	
		≥5	40.4 (34.3–46.7)	91.7 (90.8–92.5)	
		≥6	31.8 (26.1–37.9)	94.5 (93.8–95.2)	
		≥7	22.8 (17.7–28.4)	97.0 (96.4–97.5)	
		≥8	17.3 (12.8–22.5)	98.1 (97.6–98.5)	
NEWS2	0.73 (0.71–0.74)	≥3	60.4 (54.1–66.4)	76.2 (74.8–77.5)	<0.001
		≥4	49.0 (42.7–55.3)	86.2 (85.1–87.3)	
		≥5	40.8 (34.7–47.1)	91.7 (90.8–92.6)	
		≥6	32.2 (26.5–38.3)	94.6 (93.8–95.3)	
		≥7	23.5 (18.5–29.2)	97.0 (96.4–97.5)	
		≥8	18.0 (13.5–23.3)	98.1 (97.6–98.5)	

KTAS, Korean Triage and Acuity Scale; NEWS, National Early Warning Score; CI, confidence interval.

**Table 3:** Multivariate logistic regression models showing independent predictors associated with mortality and intensive care unit admission.

	Unadjusted		Model I		Model II	
	OR (95% CI)	*p*-Value	OR (95% CI)	*p*-Value	OR (95% CI)	*p*-Value
KTAS	0.17 (0.14–0.21)	<0.001	0.33 (0.26–0.42)	<0.001	0.30 (0.26–0.42)	<0.001
NEWS	1.39 (1.33–1.45)	<0.001	1.18 (1.12–1.24)	<0.001	NA	
NEWS2	1.39 (1.33–1.45)	<0.001	NA		1.18 (1.12–1.24)	<0.001
Male	1.47 (1.14–1.90)	0.003	1.30 (0.98–1.72)	0.074	1.30 (0.98–1.73)	0.072
Age, per year	1.01 (1.01–1.02)	<0.001	1.00 (1.00–1.01)	0.452	1.00 (1.00–1.01)	0.421
Medical	1.42 (0.83–2.42)	0.202	1.43 (0.78–2.62)	0.252	1.42 (0.77–2.61)	0.261
Referral route						
Outpatient department	Ref		Ref		Ref	
Direct visit	1.64 (0.88–3.04)	0.121	0.86 (0.43–1.71)	0.664	0.87 (0.44–1.72)	0.679
Transfer from another hospital	4.02 (2.11–7.65)	<0.001	1.82 (0.90–3.69)	0.095	1.84 (0.91–3.72)	0.090
Ambulance use	5.98 (4.56–7.85)	<0.001	2.71 (1.97–3.74)	<0.001	2.71 (1.97–3.74)	<0.001
Night/weekend	1.03 (0.80–1.33)	0.823	1.03 (0.76–1.38)	0.866	1.03 (0.77–1.38)	0.851
KTAS	0.17 (0.14–0.21)	<0.001	0.33 (0.26–0.42)	<0.001	0.30 (0.26–0.42)	<0.001
NEWS	1.39 (1.33–1.45)	<0.001	1.18 (1.12–1.24)	<0.001	NA	

OR, odds ratio; CI, confidence interval; KTAS, Korean Triage and Acuity Scale; NEWS, National Early Warning Score.

**Table 4:** Distribution of serious adverse events according to NEWS categories among patients triaged as KTAS levels 3 (*n* = 2760).

	SAE (+)	SAE (−)	*p*-Value
NEWS < 4	82 (72.6)	2447 (92.4)	<0.001
NEWS ≥ 5	31 (27.4)	200 (7.6)	

Data are presented as *n* (%). KTAS, Korean Triage and Acuity Scale; SAE, serious adverse event; NEWS, National Early Warning Score.

## Data Availability

The data presented in this study are available upon request from the corresponding author. The data are not publicly available due to legal restrictions.

## Supplementary Materials

The following supporting information can be downloaded at: https://www.mdpi.com/article/10.3390/diagnostics16020345/s1, Table S1: STROBE Statement—Checklist of items that should be included in reports of cohort studies.

## Abbreviations

The following abbreviations are used in this manuscript:

ED	Emergency department
SAE	Serious adverse events
ICU	Intensive Care Unit
CTAS	Canadian Triage and Acuity Scale
KTAS	Korean Triage and Acuity Scale
EWS	Early warning score
NEWS	National Early Warning Score
SpO_2_	Peripheral oxygen saturation
NEDIS	National Emergency Department Information System
ABGA	Arterial blood gas analysis
COPD	Chronic obstructive pulmonary disease
IQR	Interquartile ranges
ROC	Receiver operating characteristic
AUC	Area under the ROC
CI	Confidence intervals
